# Draft genome sequence dataset of *Actinoalloteichus caeruleus* strain 372 isolated from saline soil in Kazakhstan: genomic features and predicted biosynthetic gene clusters

**DOI:** 10.1016/j.dib.2026.113229

**Published:** 2026-09-03

**Authors:** Madina S. Alexyuk, Andrey P. Bogoyavlenskiy, Timur T. Kerimov, Vladimir E. Berezin, Pavel G. Alexyuk

**Affiliations:** Research and Production Center for Microbiology and Virology, 050010, Bogenbay batyr. Str., 105, Almaty, Kazakhstan

**Keywords:** Rare actinomycetes, Biosynthetic gene clusters, Secondary metabolites

## Abstract

This article presents the draft genome sequence of *Actinoalloteichus caeruleus* strain 372, isolated from saline soil in Kazakhstan. Whole-genome sequencing was performed using the Illumina platform, followed by de novo assembly with SPAdes. Genome quality assessment using CheckM indicated 99.25% completeness with no detectable contamination. The draft genome is 6.18 Mb in size and contains 5,024 predicted protein-coding genes, 47 tRNA genes, and 22 CRISPR arrays. Average nucleotide identity (ANI) analysis showed 96.27% identity to the closest publicly available *A. caeruleus* genome. Biosynthetic gene cluster (BGCs) analysis using antiSMASH v8.0.4 identified 31 predicted clusters, including NRPS, PKS, NRPS–PKS hybrids, terpenes, and RiPPs. These data expand the genomic resources available for the genus *Actinoalloteichus*.

Specifications Table

**Table:** 

Item	Description
Subject	Biology
Specific subject area	Microbial genomics and BGCs analysis
Type of data	Tables, figures, raw sequencing data, draft genome sequence, genome annotation
Data collection	*Actinoalloteichus caeruleus* strain 372 was isolated from saline soil in Kazakhstan. Whole-genome sequencing was performed on the Illumina MiSeq platform. Genome assembly, annotation, comparative genomic analysis, and BGCs prediction were performed using standard bioinformatics tools
Data source location	Almaty, Kazakhstan (43.24776402 N, 76.94454847 E)
Data accessibility	Direct URL to BioSample: https://www.ncbi.nlm.nih.gov/biosample/?term=SAMN55285962 Direct URL to BioProject: https://www.ncbi.nlm.nih.gov/bioproject/?term=PRJNA1421078 Direct URL to Whole Genome Data: https://www.ncbi.nlm.nih.gov/nuccore/NZ_CM143751.1 Direct URL to Raw Sequencing Data: https://www.ncbi.nlm.nih.gov/sra/?term=SRR39277614

## 1 Value of the Data

• Currently, only 11 complete genome assemblies of the genus Actinoalloteichus are available in GenBank. This dataset increases the availability of high-quality reference genomes for comparative genomic, phylogenomic, and taxonomic studies of this rare actinomycete genus.
• The dataset provides a high-quality genome assembly consisting of a single scaffold with 99.25% completeness and no detectable contamination, supporting reliable comparative genomic and functional analyses.
• Genome mining identified 31 biosynthetic gene clusters, including 29 singleton gene cluster families (GCFs), highlighting a substantial repertoire of potentially novel biosynthetic loci and providing a valuable resource for studies of secondary metabolite biosynthesis and natural product discovery.
• The availability of the genome sequence, phylogenomic analyses, and BGCs annotations enables genome-based taxonomic studies, comparative analyses within the Pseudonocardiaceae family, and further investigation of biosynthetic diversity in rare actinomycetes.

## 2 Background

Actinomycetes are an important source of secondary metabolites, but genomic resources for many rare genera remain limited [1,2]. The genus *Actinoalloteichus* is represented by a small number of publicly available genomes, restricting comparative genomic analyses and the investigation of BGCs [3].

This article presents the draft genome sequence of *Actinoalloteichus caeruleus* strain 372 isolated from saline soil in Kazakhstan. The dataset includes the genome assembly, annotation, genome quality assessment, phylogenomic characterization, comparative genomic analysis based on average nucleotide identity (ANI) and digital DNA–DNA hybridization (dDDH), and prediction of BGCs.

## 3 Data Description

*Actinoalloteichus caeruleus* strain 372 was isolated from saline soil collected in Kazakhstan and maintained as a pure culture. Whole-genome sequencing generated a high-quality draft genome of 6.18 Mb with a GC content of 72.0% (Table 1). The genome contains 5,140 predicted genes, including 5,024 protein-coding genes, 47 tRNA genes, and 22 CRISPR arrays. The assembly consisted of a single scaffold with 135 × sequencing coverage.

**Table 1:** Characteriastics of genome assembly of *Actinoalloteichus caeruleus* 372.

Characteristic	Quantity
Total genome size	6,184,314
Number of chromosomes	1
Number of scaffolds	1
Scaffold N50	6.2 Mb
Scaffold L50	1
Number of contigs	1
Contig N50	6.2 Mb
Genes (total)	5,140
Contig L50	1
GC percent	72
Genome coverage	135x
CDSs (total)	5,081
CDSs (with protein)	5,024
Genes (RNA)	59
rRNAs	3, 3, 3 (5S, 16S, 23S)
ncRNAs	3
tRNAs	47
CRISPR Arrays	22
CheckM analysis (v1.2.4)	
Completeness	99.25%
Contamination	0%

CRISPRCasTyper identified four CRISPR-Cas systems, including two type I-E loci, one type I-B locus, and one type I-C locus. A total of 22 CRISPR arrays were detected, nine of which were associated with *cas* operons.

**Fig. 1:**
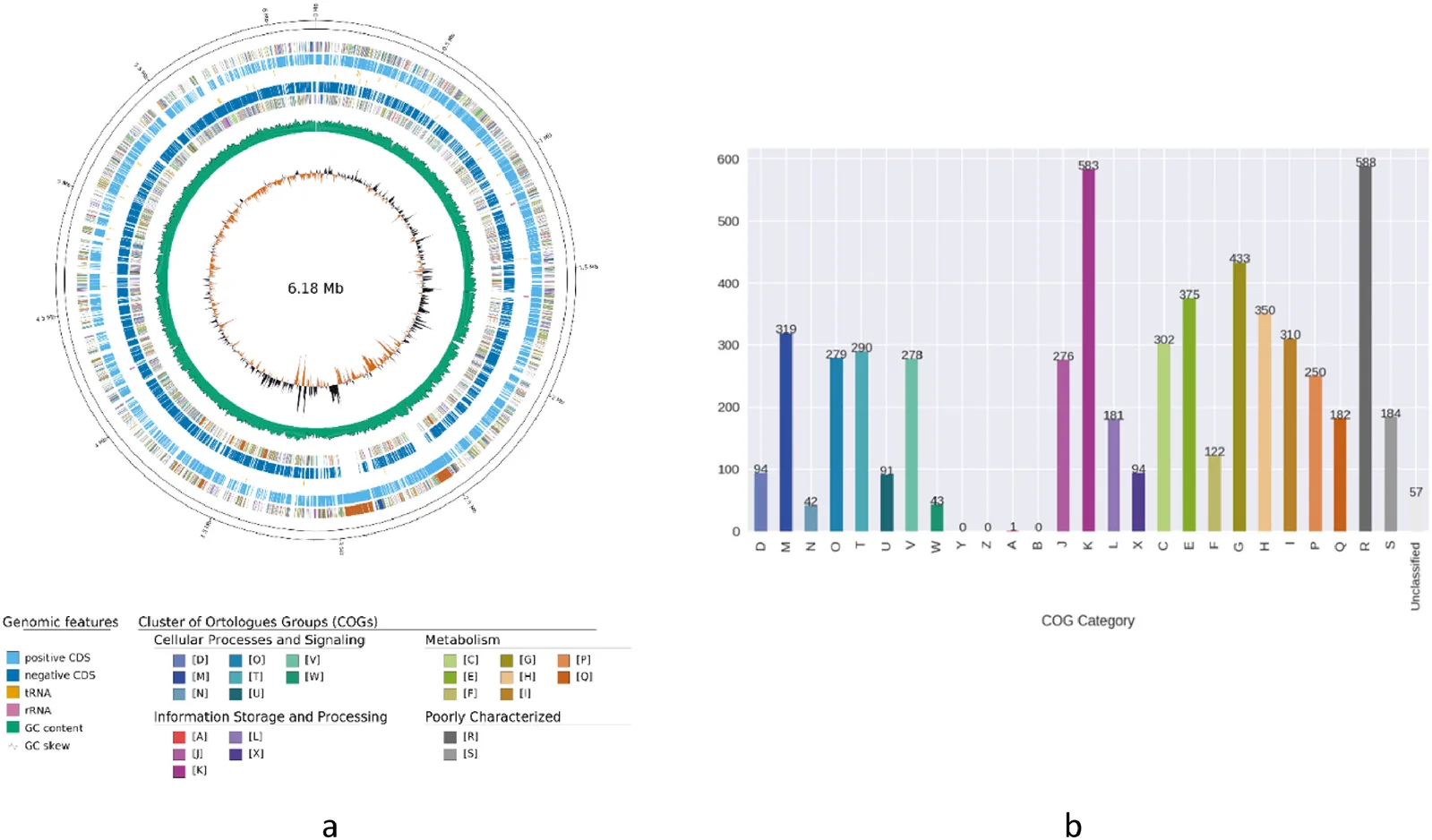
Visualization of a circular genome with calculation of GC content and COG (Cluster of Orthologs Group) functional categories; a) Location of COG functional categories in the genome; b) Quantification of COG functional categories.

Protein-coding genes were assigned to Clusters of Orthologous Groups (COG) functional categories (Fig. 1). The largest categories were general function prediction (R) and transcription (K), followed by genes involved in carbohydrate, amino acid, lipid, and energy metabolism. Genes associated with secondary metabolite biosynthesis, defense mechanisms, and mobile genetic elements were also identified.

**Fig. 2:**
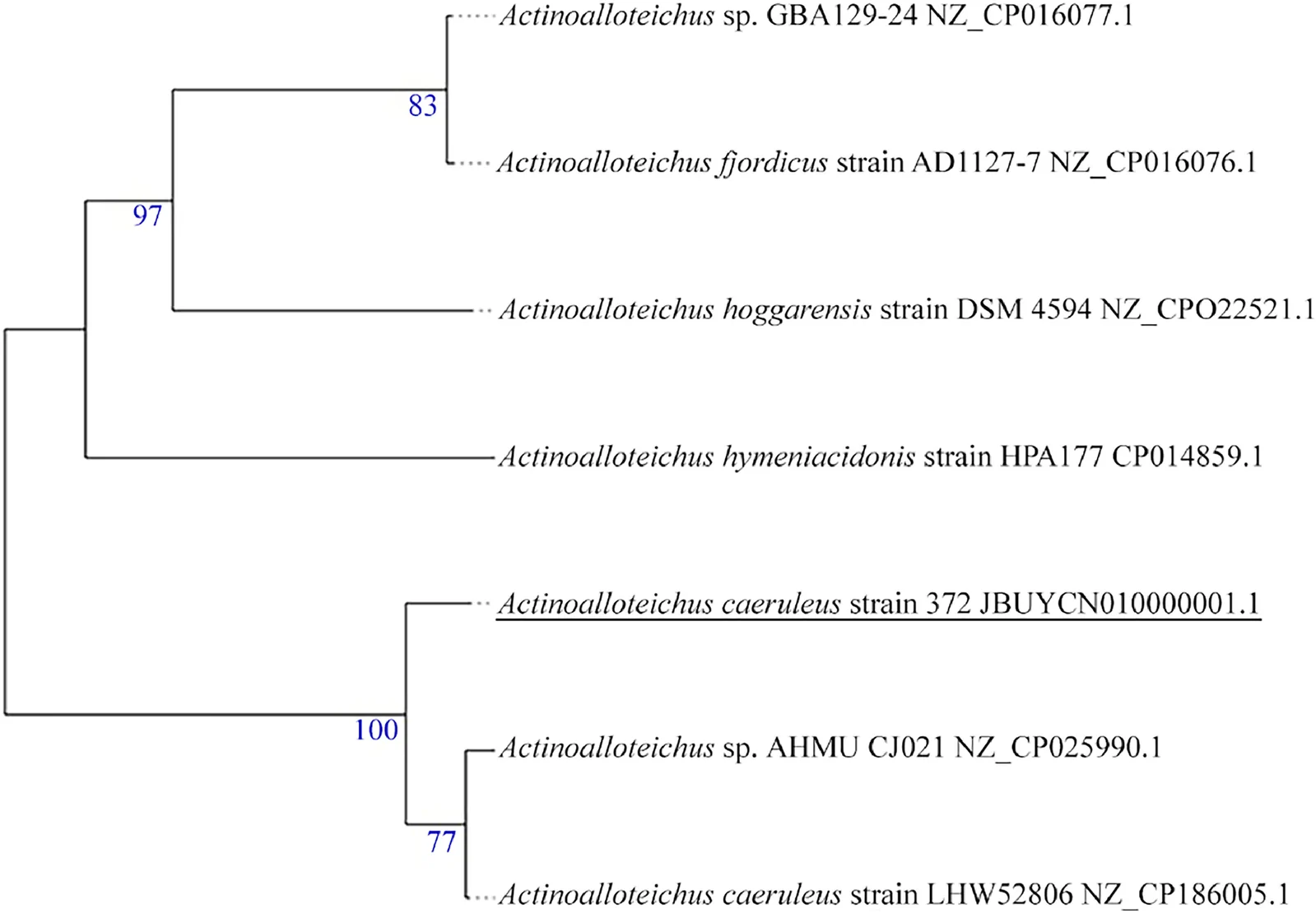
Genome-based phylogenomic tree showing the position of *Actinoalloteichus caeruleus* strain 372 among representative species of the genus *Actinoalloteichus*. The phylogenomic tree was reconstructed using the Genome BLAST Distance Phylogeny (GBDP) approach with the D5 distance formula. Node labels indicate pseudo-bootstrap support values (%) calculated from 100 replicates. Only bootstrap values greater than 60% are displayed.

Phylogenetic analysis based on the whole genome among representatives of the genera Actinoalloteichus (Fig. 2) clearly showed that the sequenced isolate clearly belongs to the group with the species caeruleus.

Phylogenomic analysis placed strain 372 within the *Actinoalloteichus caeruleus* clade (Fig. 2). Whole-genome comparisons using OrthoANI and digital DNA–DNA hybridization (dDDH) showed the highest genomic relatedness to *Actinoalloteichus* sp. AHMU CJ021 (OrthoANI, 97.23%; dDDH, 77.62%) and *Actinoalloteichus caeruleus* LHW52806 (OrthoANI, 96.71%; dDDH, 77.66%) (Table 2). These results confirmed the assignment of strain 372 to *A. caeruleus.*

**Table 2:** Results of whole genome comparison of *Actinoalloteichus caeruleus 372*.

Strain	Accession	Size (bp)	GC%	OrthoANI value (%)	dDDH (%)
*Actinoalloteichus sp.* AHMU CJ021	NZ_CP025990CP122283	6,822,750	72.5	97.23	73.9
*Actinoalloteichus caeruleus* LHW52806	NZ_CP186005	6,184,314	72.5	96.71	67.8
*Actinoalloteichus caeruleus* DSM 43889	NZ_AUBJ02000001	*6,039,370*	*72.5*	96.58	67.9

All pairwise OrthoANI and dDDH values supported the assignment of strain 372 to *Actinoalloteichus caeruleus*. Comparative genome analysis showed that members of the *A. caeruleus* clade possess the highest numbers of predicted secondary metabolite biosynthetic gene clusters among the analyzed *Actinoalloteichus* genomes (Table 3).

**Table 3:** Predicted BGCs identified by antiSMASH in genomes of *Actinoalloteichus* strains.

Accession	Organism	Number of BGCs (antiSMASH)
NZ_CP016076.1	*Actinoalloteichus fjordicus* strain ADI127-7	30
NZ_CP022521.1	*Actinoalloteichus hoggarensis* strain DSM 45943	21
NZ_CP016077.1	*Actinoalloteichus sp.* GBA129-24	28
CP014859.1	*Actinoalloteichus hymeniacidonis* strain HPA177	25
NZ_CP186005.1	*Actinoalloteichus caeruleus* strain LHW52806	31
NZ_CM143751.1	*Actinoalloteichus caeruleus* strain 372	31

The genome of *Actinoalloteichus caeruleus* strain 372 contains 31 predicted BGCs, the same number as *A. caeruleus* strain LHW52806. BGCs were predicted using antiSMASH 8.0.4 and classified according to their biosynthetic types (Table 4).

**Table 4:** Putative region clusters coding for secondary metabolites detected by antiSMASH 8.0.4 annotation of *Actinoalloteichus caeruleus 372*.

Region	Type	from	to	Similarity, %	MIBiG Accession	Short Description
1	ectoine	383,042	393,476	82.0	BGC0000853.3	ectoin (*1,4,5,6-tetrahydro-2-methyl-4-pyrimidincarboxylic acid*), C6H10N2O2 an osmoprotectant produced by *Streptomyces anulatus*.
2	lassopeptide,redox-cofactor	650,779	687,876	25.0	BGC0001483.4	Lankacidins are a class of polyketide isolated from *Streptomyces* sp. that show promising antimicrobial activity
3	terpene-precursor	1,258,687	1,279,793	39.0	BGC0000677.5	Cyclooctateen is a tricyclic diterpene (C20H34O3) with a condensed 5-8-5 ring system produced by *Streptomyces melanosporofaciens*. It acts as a potent lysophospholipase inhibitor
4	RiPP-like	1,709,647	1,720,522	30.0	BGC0001506.5	anantin B antagonist Atrial Natriuretic Factor (ANF) from *Streptomyces* sp. NRRL S-146
5	NI-siderophore	1,734,823	1,768,668	68.0	BGC0002692.2	speibonoxamine, desoxy-desferrioxamine D1, desferrioxamine D1, desferrioxamine B, didesoxy-desferrioxamine D1, desoxy-desferrioxamine B, didesoxy-desferrioxamine B from *Kribbella speibonae*
6	lanthipeptide-class-iii	1,820,430	1,843,069	52.0	BGC0000551.5	SapB 22 amino acid surfactant that is chemically related to the lantibiotic antimicrobials from *Streptomyces coelicolor* A3(2)
7	guanidinotides	1,906,043	1,928,694	58.0	BGC0002084.4	biarylitide YYH from *Planomonospora* sp. ID82291
8	lassopeptide,T2PKS	1,992,048	2,072,641	40.0	BGC0001645.3	Lagmysin Antibacterial, Herbicidal, and Plant Growth-Promoting lasso peptide from *Streptomyces* sp. NRRL S-118
9	Lanthipeptide class-ii, iii	2,127,720	2,162,282	23.0	BGC0002274.2	aspulvinone H, aspulvinone B1 butirolactonse with antiviral and anti inflammatory activity from *Aspergillus terreus* NIH2624
10	lanthipeptide-class-i	2,365,458	2,391,005	74.0	BGC0002576.2	Mechercharmycin A is a cyclic polyazole cyclopeptide of marine origin, isolated from *Thermoactinomyces* sp. YM3-251, with potent antitumor activity.
11	NRPS,T1PKS	2,393,077	2,443,693	60.0	BGC0000966.5	Caerulomycin A (CAS 21802-37-9) is a pyridine alkaloid with potent antitumor, immunosuppressive and antifungal properties. From *Actinoalloteichus* sp. WH1-2216-6
12	T1PKS,NRPS-like	2,472,727	2,565,136	86.0	BGC0001273.3	Asperlactone polyketide metabolite with primary functions as an anti-inflammatory, antifungal, antibacterial, and insecticidal agent from *Aspergillus ochraceus*
13	NRPS-like,T1PKS	2,638,109	2,747,392	95.0	BGC0002341.2	3,5-dibromo-p-anisic acid alkaloid from *Planctomycetales bacterium* 10988
14	NRPS-like,NRPS	2,829,393	2,988,245	81.0	BGC0002135.2	bovienimide A lipopeptide with insecticidal activity from *Xenorhabdus bovienii* SS-2004
15	NRPS,lanthipeptide-class-i	3,040,826	3,100,019	45.0	BGC0002576.2	Mechercharmycin A is a cyclic polyazole cyclopeptide of marine origin, isolated from *Thermoactinomyces* sp. YM3-251, with potent antitumor activity.
16	RiPP-like	3,217,703	3,228,155	40.0	BGC0001868.4	Iodinin antimicrobial peptide from *Xenorhabdus szentirmaii* DSM 16338
17	PUFA,RiPP-like,hglE-KS,terpene	3,344,120	3,407,543	60.0	BGC0000863.3	Eicosapentaenoic acid is a long-chain omega-3 polyunsaturated fatty acid), crucial for cardiovascular health from *Pseudoalteromonas* sp. DS-12
18	NI-siderophore	3,430,775	3,460,565	68.0	BGC0001478.5	desferrioxamine E siderophore with antitumor activity from *Streptomyces* sp. ID38640
19	NRPS	3,471,512	3,530,323	75.0	BGC0001132.4	Xenotetrapeptide with antimicrobial activity from *Xenorhabdus nematophila* ATCC 19061
20	NRPS	3,567,624	3,624,113	75.0	BGC0001833.4	icosalide A, icosalide B lypocyclopeptide antibiotics from *Burkholderia gladioli*
21	other,phosphonate	3,630,851	3,671,693	93.0	BGC0002608.2	ochratoxin A mycotoxin from *Aspergillus steynii*
22	NRPS,T1PKS	3,695,014	3,785,606	97.0	BGC0001128.4	gamexpeptide C oligopeptide with insecticidal activity from *Photorhabdus laumondii* subsp. *laumondii* TTO1
23	terpene	3,852,650	3,873,816	44.0	BGC0002150.2	Triculamin antimycobacterial activity from *Streptomyces triculaminicus*
24	terpene-precursor	4,078,270	4,099,310	49.0	BGC0002736.2	preaspterpenacid I sesquiterpene from *Aspergillus terreus* NIH2624
25	NRPS-like,arylpolyene	4,526,103	4,569,020	55.0	BGC0001641.4	Kolossin antinematode peptide from *Photorhabdus laumondii* subsp. *laumondii* TTO1
26	hglE-KS	4,658,753	4,704,290	60.0	BGC0001273.3	Asperlactone polyketide metabolite with primary functions as an anti-inflammatory, antifungal, antibacterial, and insecticidal agent from *Aspergillus ochraceus*
27	lanthipeptide-class-i	4,743,259	4,767,466	44.0	BGC0001289.5	Penisin novel class Ia lantibiotic antimicrobial peptide produced by *Paenibacillus ehimensis*, which acts as an effective agent against MRSA. It is a bacteriocin-like peptide with a random coil structure, containing six thioether cross-links
28	terpene	4,879,612	4,900,502	45.0	BGC0000587.5	microcin H47 peptide antibiotic from *Escherichia coli*
29	RRE-containing	5,070,740	5,091,000	54.0	BGC0002631.2	Myxarylin biarylitide MeYLH from *Pyxidicoccus fallax*
30	lassopeptide	6,022,766	6,044,778	52.0	BGC0001493.5	Achromosin antimicrobial lasso peptide from *Streptomyces achromogenes* subsp. *achromogenes*
31	lassopeptide	6,078,510	6,101,085	68.0	BGC0002006.2	Albusnodin is a specialized acetylated lassopeptide produced by *Streptomyces albus*. Its primary function is believed to be a resistance mechanism that protects the producing cells from self-poisoning.

BiG-SCAPE analysis classified the 31 predicted biosynthetic gene clusters into gene cluster families (GCFs) using a distance cutoff of 0.3 (Table 5). Two BGCs (Regions 1 and 11) were assigned to multi-member GCFs containing MIBiG reference biosynthetic gene clusters, whereas the remaining 29 BGCs were classified as singleton GCFs because they did not cluster with either other predicted BGCs or MIBiG reference BGCs at the selected threshold. The singleton GCFs comprised diverse biosynthetic categories, including RiPP, terpene, NRPS, PKS, NRPS/PKS hybrids, NRPS/RiPP, PKS/RiPP, PKS/RiPP/terpene, and other BGC types.

**Table 5:** Classification of predicted biosynthetic gene clusters (BGCs) into gene cluster families (GCFs) based on BiG-SCAPE analysis (distance cutoff = 0.3).

BGC category	Multi-member GCFs (clustered with MIBiG reference BGCs)	Singleton GCFs
NRPS	—	Region 14 (NRPS-like, NRPS); Region 19 (NRPS); Region 20 (NRPS)
NRPS/PKS	Region 11 (NRPS, T1PKS)	Region 12 (T1PKS, NRPS-like); Region 13 (NRPS-like, T1PKS); Region 22 (NRPS, T1PKS); Region 25 (NRPS-like, arylpolyene)
NRPS/RiPP	—	Region 15 (NRPS, lanthipeptide class I)
Other	Region 1 (ectoine)	Region 5 (NIS-siderophore); Region 18 (NIS-siderophore); Region 21 (phosphonate)
PKS	—	Region 26 (hglE-KS)
PKS/RiPP	—	Region 8 (lassopeptide, T2PKS)
PKS/RiPP/Terpene	—	Region 17 (PUFA, RiPP-like, hglE-KS, terpene)
RiPP	—	Region 2 (lassopeptide, redox-cofactor); Region 4 (RiPP-like); Region 6 (lanthipeptide class III); Region 7 (guanidinotides); Region 9 (lanthipeptide class I/III); Region 10 (lanthipeptide class I); Region 16 (RiPP-like); Region 27 (lanthipeptide class I); Region 29 (RRE-containing); Region 30 (lassopeptide); Region 31 (lassopeptide)
Terpene	—	Region 3 (terpene-precursor); Region 23 (terpene); Region 24 (terpene-precursor); Region 28 (terpene)

## 4 Experimental Design, Materials and Methods

Soil samples were collected from a saline takyr soil in the Ili–Balkhash region, Kazakhstan (45.031850, 75.722234). *Actinoalloteichus caeruleus* strain 372 was isolated using standard microbiological techniques and preserved in 20% (v/v) glycerol at −80°C [4].

Genomic DNA was extracted using the PureLink Genomic DNA Mini Kit (Thermo Fisher Scientific, USA). Sequencing libraries were prepared with the Nextera XT DNA Library Preparation Kit (Illumina, USA) and sequenced on the Illumina MiSeq platform (2 × 300 bp) [5]. Raw reads were quality filtered using FastQC (Galaxy Version 0.74) and Trimmomatic (Galaxy Version 0.38.1) [6]. Raw sequencing reads were evaluated using FastQC (v0.11.9). Read trimming was performed with Trimmomatic (v0.39) using the following parameters: adapter removal (ILLUMINACLIP), leading and trailing base trimming with a quality threshold of 20 (LEADING:20, TRAILING:20), sliding window quality trimming (SLIDINGWINDOW:4:20), and a minimum read length of 50 bp (MINLEN:50). Reads assembled de novo using SPAdes 3.12.0, Tadpole 38.84, MEGAHIT 1.2.9, and Geneious Assembler v 1.3.2. Unless otherwise specified, all software was run using the default parameters. The final genome assembly was obtained by integrating and comparing the results generated by different assembly software packages, resulting in a consensus assembly with improved completeness and accuracy

Genome annotation was performed using the NCBI Prokaryotic Genome Annotation Pipeline (PGAP) [7]. Phylogenomic analysis was conducted using TYGS [8,9], while genomic relatedness was assessed using OrthoANI [10] and the Genome-to-Genome Distance Calculator (GGDC) [11]. CRISPR-Cas systems were identified using CRISPRCasTyper [12], and functional annotation was performed using eggNOG/COG [13]. BGCs were predicted with antiSMASH v8.0.4 [14]. Sequence similarity analysis and gene cluster family (GCF) assignment were performed using BiG-SCAPE v2.0 [15] with the default global alignment mode and a GCF distance cutoff of 0.3. Predicted BGCs that did not cluster with either other predicted BGCs or MIBiG reference BGCs at this threshold were classified as singleton GCFs. Circular genome visualization was generated using GenoVi 0.2.16 [16].

Raw sequencing data are available in the NCBI Sequence Read Archive under accession BioProject: PRJNA1421078; BioSample: SAMN55285962; GenBank: NZ_CM143751 [17]; SRA: SRR39277614.

## Limitations

The biosynthetic gene clusters were predicted using antiSMASH v8.0.4 and were not experimentally validated. The dataset is based on a single *Actinoalloteichus caeruleus* strain and does not represent intraspecies genomic diversity.

## Ethics Statement

Authors have read and follow the ethical requirements for publication in Data in Brief and confirming that the current work does not involve human subjects, animal experiments, or any data collected from social media platforms.

## Credit Author Statement

**Madina S. Alexyuk:** Methodology, Validation, Formal analysis, Investigation, Writing - Original Draft, Visualization, Writing - Review & Editing. **Andrey P. Bogoyavlenskiy:** Conceptualization, Formal analysis, Data Curation, Writing - Original Draft, Visualization, Writing - Review & Editing. **Timur T. Kerimov:** Software, Data Curation, Writing - Review & Editing. **Vladimir E. Berezin:** Supervision, Resources, Writing - Review & Editing. **Pavel G. Alexyuk**: Software, Data Curation, Writing - Review & Editing.
